# Luteolin Potentially Alleviates Methamphetamine Withdrawal-Induced Negative Emotions and Cognitive Deficits Through the AKT/FOXO1/HO-1 Signaling Pathway in the Prefrontal Cortex and Caudate Putamen

**DOI:** 10.3390/ijms26125739

**Published:** 2025-06-15

**Authors:** Baoyao Gao, Ran An, Min Liang, Xinglin Wang, Jianhang Peng, Xingyao Chen, Zijun Liu, Tao Li, Xinshe Liu, Jianbo Zhang, Wei Han

**Affiliations:** 1 College of Forensic Medicine, Xi’an Jiaotong University, Xi’an 710061, China; gby19960908@stu.xjtu.edu.cn (B.G.); anr_ann@stu.xjtu.edu.cn (R.A.); liangmin0526@stu.xjtu.edu.cn (M.L.); q1225426987@stu.xjtu.edu.cn (X.W.); pjh980513@stu.xjtu.edu.cn (J.P.); syachan@stu.xjtu.edu.cn (X.C.); lzijun@stu.xjtu.edu.cn (Z.L.); litao050428@xjtu.edu.cn (T.L.); lxins@mail.xjtu.edu.cn (X.L.); 2 Key Laboratory of National Health Commission for Forensic Science, Xi’an Jiaotong University, Xi’an 710061, China

**Keywords:** methamphetamine withdrawal, negative emotions, cognitive deficits, luteolin, protein kinase B (AKT), forkhead box protein 1 (FOXO1), heme-oxygenase-1 (HO-1)

## Abstract

Methamphetamine (METH) misuse-induced affective and cognitive dysfunctions cause severe global health and economic burdens. However, the mechanisms underlying METH withdrawal-induced negative emotions and cognitive deficits, as well as the treatment strategies for them, remain elusive. Previous findings suggest that METH use triggers neuroinflammation and neuronal apoptosis, and protein kinase B (AKT), forkhead box protein 1 (FOXO1), and heme-oxygenase-1 (HO-1) are implicated in these processes. In the present study, we aimed to reveal the role and potential mechanisms of luteolin, a flavonoid phytochemical with anti-inflammatory and antioxidative properties, in METH withdrawal-induced negative emotions and cognitive deficits. We found that prolonged METH withdrawal led to an increase in neuronal activity and a decrease in the protein expression of phosphorylated AKT (p-AKT) and HO-1 in the prefrontal cortex (PFC) and caudate putamen (CPu). Luteolin pretreatment partially mitigated these METH withdrawal-induced negative emotions and cognitive deficits, and prevented the abnormal activation of PFC and CPu as well as the downregulation of AKT/HO-1 expression. Notably, we further observed that luteolin inhibited the METH-induced nuclear translocation of FOXO1. Our findings suggest that luteolin may alleviate METH withdrawal-induced affective and cognitive dysfunctions by reducing oxidative injury in the brain through the AKT/FOXO1/HO-1 pathway, highlighting its potential for treating drug addiction-related health issues.

## 1. Introduction

Amphetamines are among the most widely misused illicit drugs globally, with over 30 million users according to the World Drug Report 2024. Methamphetamine (METH), a typical amphetamine, is the most misused drug in China [1]. As a potent psychostimulant that can cross the blood–brain barrier (BBB) [2], METH exhibits severe neurotoxicity, partly due to oxidative stress injury and neuroinflammation in the brain [3,4,5]. Prolonged METH use can cause addiction and cognitive deficits [6,7]. Moreover, patients often develop psychological disorders during METH withdrawal, particularly anxiety and depression [8,9]. Previous studies have demonstrated that approximately 39–64% of METH users exhibit depressive symptoms [10,11]. However, the precise mechanisms underlying METH withdrawal-induced negative emotions and cognitive deficits remain unclear.

Chronic METH use triggers various neural adaptations, including neuronal plasticity and gene expression changes in various brain regions, such as the prefrontal cortex (PFC) [12,13] and striatum [14,15]. The PFC is associated with cognitive dysfunction after repeated drug use [16], while the striatum [17] is mainly implicated in the rewarding effects of addictive substances. In the PFC, the inhibitory neurons are activated and excitatory neurons are suppressed to facilitate METH-associated memory retrieval [18]. METH exposure has been shown to increase neuronal activity in the dorsal striatum (also known as the caudate putamen, CPu) [19]. Although mounting evidence has revealed neural circuit and neuronal plasticity changes in multiple brain regions during METH addiction [20,21,22], the activity changes and potential roles of neurons in the CPu and PFC in METH withdrawal-induced negative emotions require further exploration.

Gene expression dynamics are crucial in neural plasticity alterations during substance addiction [23]. Chronic METH use has been shown to decrease the expression of heme-oxygenase-1 (*HO-1*), a cytoprotective gene protecting cells from METH-induced oxidative stress injury and apoptosis [24,25]. Protein kinase B (AKT) is involved in cell proliferation, differentiation, and survival [26]. The phosphorylation of AKT facilitates the expression of HO-1 [25,27]. Forkhead box protein 1 (FOXO1) is one kind of transcription factor associated with neuroinflammation and apoptosis. Intracerebral hemorrhage has been shown to cause neuroinflammation, neuronal apoptosis, and the activation of FOXO1 [28]. The inhibition of the phosphoinositide 3-kinase (PI3K)/AKT pathway upregulates FOXO1 expression [29]. Thus, we hypothesize that the AKT/FOXO1/HO-1 signaling pathway in the PFC and CPu may play a role in METH withdrawal-induced negative emotions and cognitive deficits.

Recently, food-derived phytochemicals and their derivatives have shown benefits to brain function [30,31]. Luteolin, a flavonoid, has demonstrated neuroprotective effects in cadmium or ischemic/reperfusion-induced brain injuries [32,33]. It can enhance cell viability and downregulate apoptosis via the PI3K/AKT signaling pathway, thereby ameliorating ischemic stroke [34]. Moreover, luteolin can cross the BBB and protect neurons from apoptosis by inhibiting neuroinflammation and oxidative stress [35,36]. However, the role and molecular mechanisms of luteolin in METH withdrawal-induced affective and cognitive dysfunctions are still unknown.

In this study, we first examined METH withdrawal-induced negative emotions, cognitive deficits, and AKT/HO-1 signaling expression changes in the PFC and CPu. Neuronal activity alterations in these brain regions after prolonged METH withdrawal were also detected. Then, we investigated the effects of luteolin on METH withdrawal-induced negative emotions and cognitive deficits. The AKT/HO-1 expression and neuronal activity alterations in METH withdrawal after luteolin treatment were further determined. Finally, the dynamics of nuclear translocation of FOXO1 were examined to reveal the potential mechanisms involved in the protective role of luteolin in METH withdrawal-induced affective and cognitive dysfunctions.

## 2. Results

### 2.1. METH Withdrawal Induces Anxiety and Depressive-like Behaviors, as Well as Cognitive Deficits

**Figure 1:**
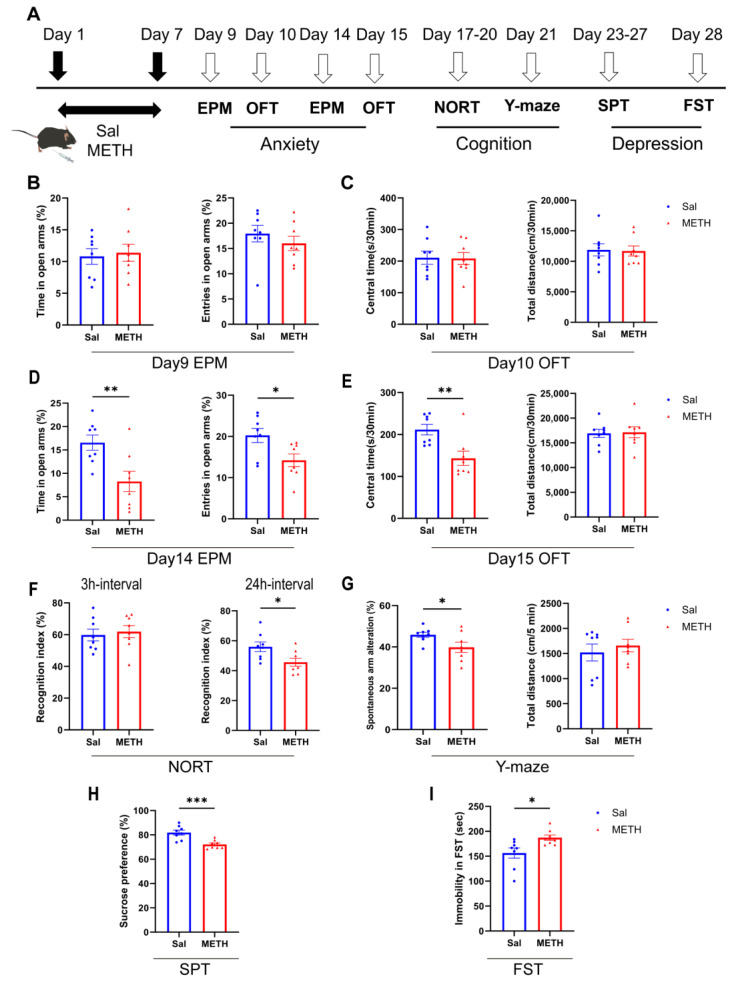
METH withdrawal-induced negative emotions and cognitive deficits. (**A**) Experimental procedure. (**B**–**E**) Anxiety-like behaviors. (**B**,**D**) Time spent and number of entries in the open arm (%) of the EPM test. (**C**,**E**) Time spent in the central zone (%) and total distance traveled in the OFT. (**F**,**G**) Cognitive function (episodic and spatial memories) test. (**F**) Time spent accessing the new object (%) in the NORT. (**G**) Spontaneous alternations (%) in the Y-maze test. (**H**,**I**) Depressive-like behaviors. (**H**) Sucrose consumption (%) in the sucrose preference test. (**I**) Immobility time in the FST. Data are expressed as the mean ± SEM; *n* = 8/group. *: *p* < 0.05, **: *p* < 0.01, ***: *p* < 0.001 compared to the Sal group. Sal, saline; METH, methamphetamine; EPM, elevated plus maze; OFT, open field test; NORT, novel object recognition test; SPT, sucrose preference test; FST, forced swim test.

We first examined METH withdrawal-induced affective and cognitive function changes (Figure 1A). Our results revealed that mice did not exhibit anxiety-like behaviors 48–72 h after METH withdrawal (on days 9 and 10, Figure 1B,C). However, they showed significant anxiety-like behaviors one week post-withdrawal. Compared to the saline (Sal) group, the METH group mice spent less time in and showed decreased entries into the open arm of the EPM, and spent less time in the central zone of the OFT (Figure 1D,E). In the NORT, the METH group mice exhibited a decreased recognition index compared to the Sal group in the 24 h interval test, but not for the 3 h interval test (Figure 1F). Additionally, the METH group displayed fewer spontaneous alterations in the Y-maze test, without alterations in the total distance traveled (Figure 1G). Regarding depressive-like behaviors, the METH group mice showed a reduced sucrose preference (Figure 1H) and an increased immobility time in the FST (Figure 1I), compared to the Sal group. These findings demonstrate that METH withdrawal precipitates negative emotions and cognitive deficits.

### 2.2. METH Withdrawal Increases the Neuronal Activity in the PFC and CPu

**Figure 2:**
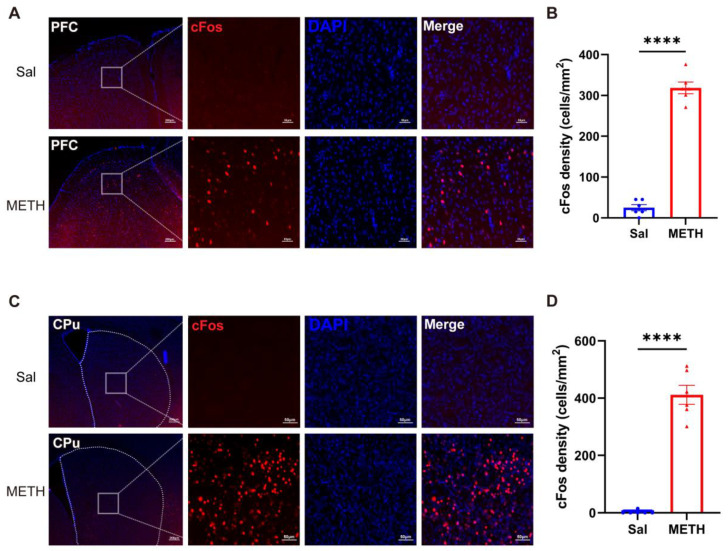
METH withdrawal activated c-Fos expression in the PFC and CPu. (**A**,**B**) Representative images of c-Fos staining and quantitative analysis of the density of c-Fos positive cells in the PFC. (**C**,**D**) Representative images of c-Fos staining and quantitative analysis of the density of c-Fos-positive cells in the CPu. (**A**,**C**). Scale bars represent 200 μm under low magnification and 50 μm under high magnification. Data are presented as the mean ± SEM; *n* = 3/group, two slices per mouse. ****: *p* < 0.0001 compared to the Sal group. Sal, saline; METH, methamphetamine; PFC, prefrontal cortex; CPu, caudate putamen.

To reveal the METH withdrawal-induced activity changes in neurons in the PFC and CPu, we detected and compared the differences in the c-Fos-positive cell densities in the two brain regions. After the final behavioral test, the mice were immediately sacrificed for tissue collection. The c-Fos-positive cell densities exhibited a significant increase in both the PFC (Figure 2A,B) and CPu (Figure 2C,D) in the METH group compared to the Sal group, demonstrating that prolonged METH withdrawal increased the neuronal activity in these two brain regions.

### 2.3. METH Withdrawal Downregulates p-AKT and HO-1 Expression in the PFC and CPu

**Figure 3:**
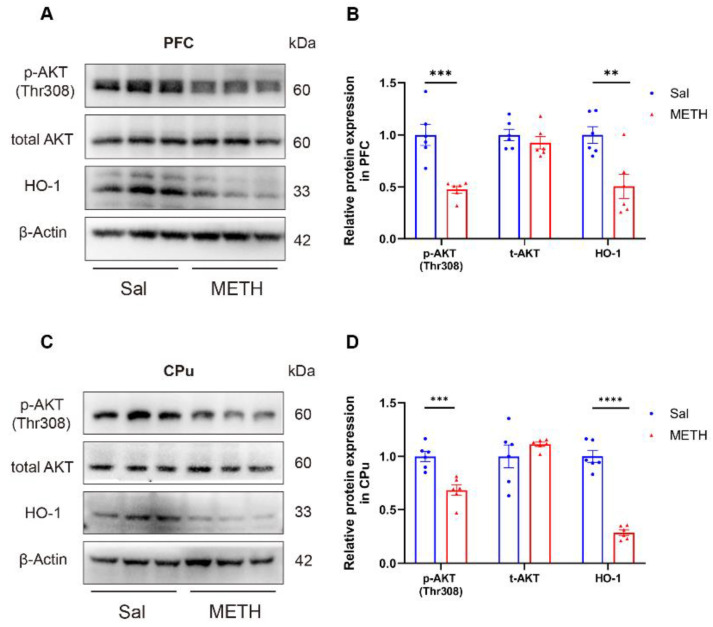
METH withdrawal downregulated p-AKT and HO-1 expression in the PFC and CPu. (**A**,**B**) Protein levels of p-AKT (Thr308), total AKT (t-AKT), HO-1, and β-actin in the PFC. (**C**,**D**) Protein levels of p-AKT(Thr308), t-AKT, HO-1, and β-actin in the CPu. Data are presented as the mean ± SEM; *n* = 6/group. **: *p* < 0.01, ***: *p* < 0.001, ****: *p* < 0.0001 compared to the Sal group. Sal, saline; METH, methamphetamine; PFC, prefrontal cortex; CPu, caudate putamen.

To investigate the potential molecular mechanisms underlying METH withdrawal-induced negative emotions and cognitive deficits, we measured the AKT and HO-1 protein expression in the PFC and CPu using Western blot analysis. Our results revealed that the p-AKT and HO-1 protein levels were significantly decreased in the PFC (Figure 3A,B) and CPu (Figure 3C,D) of the METH-exposed mice compared to the Sal-exposed mice, while there was no difference in the total AKT expression between the groups in both regions. These findings suggest that the downregulation of p-AKT and HO-1 expression in both the PFC and CPu may be associated with METH withdrawal-induced negative emotions and cognitive deficits.

### 2.4. The Luteolin Pretreatment Potentially Alleviates METH Withdrawal-Induced Anxiety and Depressive-like Behaviors, as Well as Cognitive Deficits

**Figure 4:**
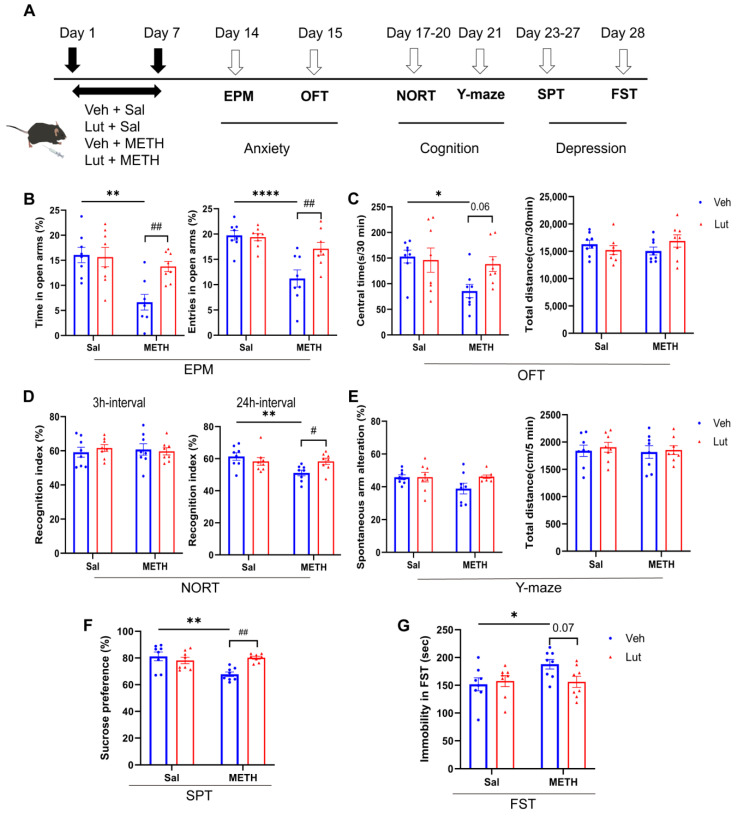
The luteolin pretreatment alleviated METH withdrawal-induced negative emotions and cognitive deficits. (**A**) Experimental procedure. (**B**,**C**) Anxiety-like behaviors. (**B**) Time spent and number of entries in the open arm (%) in the EPM test. (**C**) Time spent in the central zone (%) and total distance traveled in the OFT. (**D,E**) Cognitive function test. (**D**) Time spent accessing the new object (%) in the NORT. (**E**) Spontaneous alternations (%) in the Y-maze test. (**F**,**G**) Depressive-like behaviors. (**F**) Sucrose consumption (%) in the sucrose preference test. (**G**) Immobility time in the FST. Data are expressed as the mean ± SEM; *n* = 8/group. *: *p* < 0.05, **: *p* < 0.01, ****: *p* < 0.0001 compared to the Sal group; #: *p* < 0.05, ##: *p* < 0.01 compared to the Veh + METH group. Sal, saline; Veh, vehicle; Lut, luteolin; METH, methamphetamine; EPM, elevated plus maze; OFT, open field test; NORT, novel object recognition test; SPT, sucrose preference test; FST, forced swim test.

To examine the effects of luteolin on METH withdrawal-induced affective and cognitive dysfunctions, we pretreated the mice with luteolin 30 min before METH administration and assessed the changes in METH withdrawal-induced anxiety and depressive-like behaviors, as well as cognitive functions (Figure 4A). For anxiety-like behaviors, the luteolin pretreatment reversed the METH withdrawal-induced decrease in time spent in and in the number of entries into the open arms of the EPM (Figure 4B). There was also a trend that showed an increase in the time spent in the central area of the OFT in the luteolin + METH group compared to the vehicle + METH (Veh + METH) group (Figure 4C). Regarding cognitive function, the luteolin treatment alleviated the METH withdrawal-induced decrease in the recognition index in the 24 h interval NORT, but not in the 3 h interval test (Figure 4D). However, no difference in spontaneous alterations was found among groups in the Y-maze test (Figure 4E). Similarly, for depression-like behaviors, the luteolin treatment prevented the METH withdrawal-induced decrease in sucrose preference in SPT (Figure 4F). Meanwhile, there was a trend of decreasing immobility time in the luteolin + METH group compared to the Veh + METH group in the FST (Figure 4G). Collectively, these results suggest that luteolin pretreatment partially alleviates METH withdrawal-induced anxiety and depressive-like behaviors, as well as cognitive deficits.

### 2.5. Luteolin Attenuates the METH Withdrawal-Induced Abnormal Activation of Neurons in the PFC and CPu

**Figure 5:**
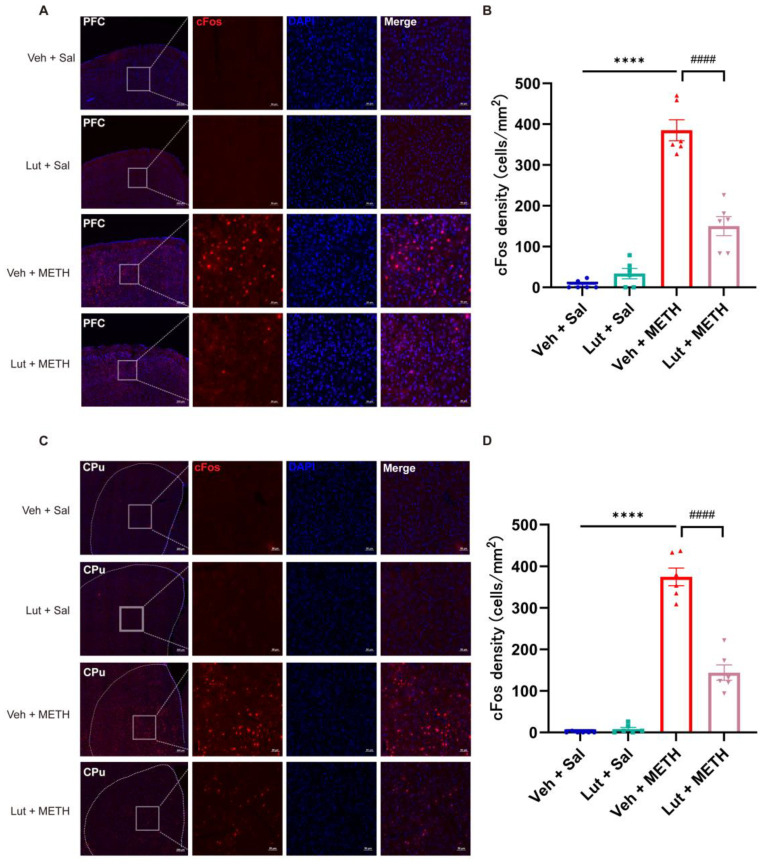
Luteolin pretreatment downregulated the METH withdrawal-induced activation of c-Fos expression in the PFC and CPu. (**A**,**B**) Representative images of c-Fos staining and quantitative analysis of the density of c-Fos-positive cells in the PFC. (**C**,**D**) Representative images of c-Fos staining and quantitative analysis of the density of c-Fos positive cells in the CPu. (**A**,**C**) Scale bars represent 200 μm under low magnification and 50 μm under high magnification. Data are presented as the mean ± SEM; *n* = 3/group, two slices per mouse. ****: *p* < 0.0001, compared to the Veh + Sal group; ####: *p* < 0.0001 compared to the Lut + METH group. Sal, saline; METH, methamphetamine; Veh, vehicle; Lut, luteolin; PFC, prefrontal cortex; CPu, caudate putamen.

To verify the correlations between the luteolin-induced alleviation of METH withdrawal-associated negative affective/cognitive dysfunctions and neuronal activity changes in the PFC and CPu, we measured the c-Fos expression in these two brain regions following the behavioral tests. We found that luteolin treatment inhibited the METH withdrawal-induced activation of neurons in the PFC (Figure 5A,B) and CPu (Figure 5C,D), demonstrating decreased c-Fos-positive cell densities in the luteolin + METH group compared to the Veh + METH group. These results indicate that the protective effects of luteolin on METH withdrawal-induced negative emotions and cognitive deficits may be associated with a downregulation of abnormal neuronal activation in the PFC and CPu.

### 2.6. Luteolin Prevents the METH Withdrawal-Induced Downregulation of p-AKT and HO-1 Expression in the PFC and CPu

**Figure 6:**
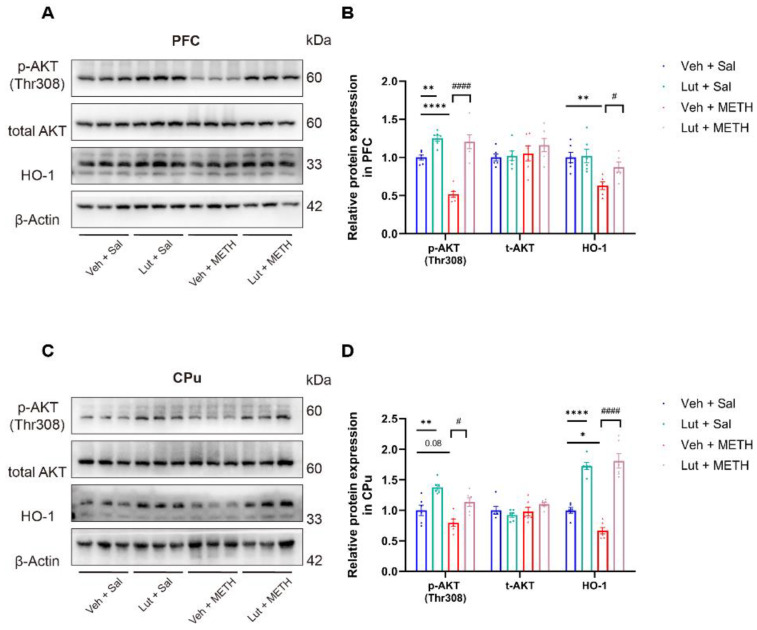
Luteolin pretreatment reversed the METH withdrawal-induced downregulation of p-AKT and HO-1 in the PFC and CPu. (**A**,**B**) Protein levels of p-AKT(Thr308), total AKT (t-AKT), HO-1, and β-actin in the PFC. (**C**,**D**) Protein levels of p-AKT(Thr308), t-AKT, HO-1, and β-actin in the CPu. Data are presented as the mean ± SEM; *n* = 6/group. *: *p* < 0.05, **: *p* < 0.01, ****: *p* < 0.0001 compared to the Veh + Sal group; #: *p* < 0.05, ####: *p* < 0.0001 compared to the Lut + METH group. Sal, saline; METH, methamphetamine; Veh, vehicle; Lut, luteolin; PFC, prefrontal cortex; CPu, caudate putamen.

To elucidate the potential molecular mechanisms underlying the effects of luteolin on METH withdrawal-induced negative emotions and cognitive deficits, we examined the AKT and HO-1 expression level in the PFC and CPu. Our results demonstrated that luteolin pretreatment reversed the METH withdrawal-induced downregulation of p-AKT and HO-1 expression in the PFC (Figure 6A,B) and CPu (Figure 6C,D). These findings suggest that luteolin may exert a protective effect on METH withdrawal-induced affective and cognitive dysfunctions by potentially activating AKT/HO-1 signaling in the PFC and CPu.

### 2.7. Luteolin Inhibits METH Withdrawal-Induced Nuclear Translocation of FOXO1

**Figure 7:**
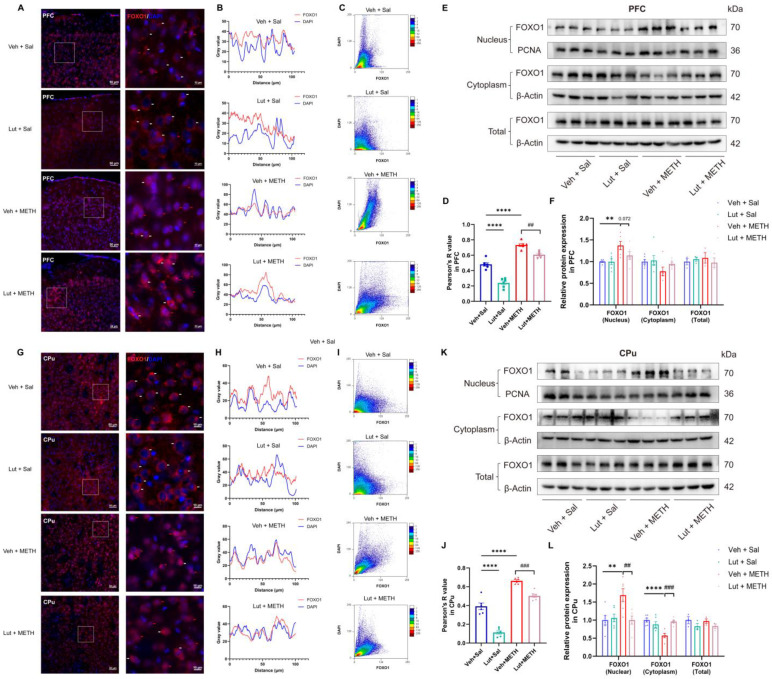
Luteolin pretreatment prevented the METH withdrawal-induced activation of FOXO1 in the PFC and CPu. (**A**) FOXO1 immunofluorescence in the PFC. (**B**) Colocalization curve plot of FOXO1 and DAPI in the PFC. (**C**) Scatter plot showing the fluorescence colocalization of FOXO1 and the nucleus in the PFC. (**D**). Pearson’s correlation for the colocalization of FOXO1 and DAPI in the PFC. (**E**) Representative immunoblot images of FOXO1 expression in the PFC. (**F**) Comparisons of relative protein expression levels of FOXO1 among groups in the PFC. (**G**) FOXO1 immunofluorescence in the CPu. (**H**) Colocalization curve plot of FOXO1 and DAPI in the CPu. (**I**) Scatter plot showing the fluorescence colocalization of FOXO1 and the nucleus in the CPu. (**J**). Pearson’s correlation for the colocalization of FOXO1 and DAPI in the CPu. (**K**) Representative immunoblot images of FOXO1 expression in the CPu. (**L**) Comparisons of relative protein expression level of FOXO1 among groups in the CPu. (**A**,**G**) Scale bars represent 50 μm under low magnification and 10 μm under high magnification; *n* = 3/group, two slices per mouse. Red arrows represent nuclear translocation and white arrows represent the cytoplasm localization of FOXO1. (**F**,**L**) Data are presented as the mean ± SEM; *n* = 6/group for nucleus/cytoplasm FOXO1; *n* = 3/group for total FOXO1. **: *p* < 0.01, ****: *p* < 0.0001 compared to the Veh + Sal group; ##: *p* < 0.01, ###: *p* < 0.001 compared to the Lut + METH group. Sal, saline; METH, methamphetamine; Veh, vehicle; Lut, luteolin; PFC, prefrontal cortex; CPu, caudate putamen.

We further investigated the FOXO1 expression dynamics in the PFC (Figure 7A–F) and CPu (Figure 7G–L) following the luteolin pretreatment. Immunofluorescence analysis demonstrated that prolonged METH withdrawal led to an accumulation of FOXO1 in the nucleus of neural cells, suggesting the activation of FOXO1. The luteolin pretreatment could inhibit the nuclear accumulation of FOXO1 in both the PFC (Figure 7A) and CPu (Figure 7G). Next, by using fluorescence colocalization, we found that the fluorescence signals of FOXO1 and the nucleus exhibited strong spatial overlap in the Veh + METH group in both the PFC and CPu (Figure 7B,H). The scatter plot also showed a linear distribution of points along the diagonal in the Veh + METH group in these two brain regions (Figure 7C,I). The above results indicate that METH withdrawal induces colocalization between FOXO1 and the nucleus, suggesting the nuclear translocation of FOXO1. Notably, luteolin pretreatment could attenuate the METH withdrawal-induced nuclear translocation of FOXO1 in both the PFC and CPu. Pearson’s correlation analysis revealed a significant interaction between METH withdrawal and the luteolin treatment, with a marked decrease in the R value in the luteolin + METH group compared to the Veh + METH group (Figure 7D,J). Additionally, the Western blotting analysis of nuclear, cytoplasmic, and total FOXO1 levels further validated that the luteolin treatment mitigated the METH withdrawal-induced increase in nuclear FOXO1 expression (Figure 7E,F,K,L). Overall, our findings suggest that the luteolin pretreatment attenuates the METH withdrawal-induced activation of FOXO1 by inhibiting its nuclear translocation.

## 3. Discussion

METH withdrawal-induced negative emotions and cognitive deficits cause substantial health and economic burdens to society worldwide [37,38]. However, the underlying mechanisms remain poorly understood, and effective treatments are limited. In the present study, we investigated the role and potential mechanisms of luteolin in METH withdrawal-induced affective and cognitive dysfunctions. Our results demonstrated that METH withdrawal led to anxiety and depressive-like behaviors, as well as cognitive deficits. Neuronal activity was increased and p-AKT/HO-1 expression was decreased in the PFC and CPu after prolonged METH withdrawal. Interestingly, luteolin pretreatment could partially ameliorate METH withdrawal-induced affective and cognitive dysfunctions, and reverse METH withdrawal-induced abnormal neuron activation, as well as the downregulation of p-AKT/HO-1 signaling. The potentially protective effects of luteolin on METH-induced affective and cognitive dysfunctions may be attributable to the AKT-mediated activation of HO-1 expression and to a decrease in the nuclear translocation of transcription factor FOXO1.

Neuronal activity alterations are critical for neural plasticity in both physiological and pathological conditions, such as aging and drug addiction [39,40]. METH use has been shown to increase neuronal activity, demonstrated as an increase in c-Fos expression in the orbitofrontal cortex (OFC) and dorsal striatum (CPu) [19,41]. We consistently found that METH withdrawal led to the activation of brain regions in the PFC and CPu. The PFC and CPu have long been implicated in addictive drug-induced rewarding and reinforcing effects [19,42]. However, few studies have investigated their roles in METH withdrawal-induced affective dysfunctions. Interestingly, our findings suggest that neuronal activation in the PFC and CPu may be associated with METH withdrawal-induced negative emotions, offering new insights into their potential roles in drug addiction.

Beyond neuronal activity, gene expression changes also represent long-term neural adaptations following METH exposure [43]. We observed that the expression levels of p-AKT and HO-1 were decreased after prolonged METH withdrawal. The AKT-induced activation of HO-1 is known for its antioxidative properties [25,44]. Previous findings have indicated that acute METH exposure increases PI3K/AKT [45] and HO-1 [46] expression, demonstrating compensatory and protective mechanisms to the increased oxidative stress and apoptosis in the brain caused by METH. In accordance with our findings, chronic METH use has been shown to downregulate AKT and HO-1 expression [25]. Hence, we speculate that the prolonged METH withdrawal-induced downregulation of p-AKT and HO-1 observed in our study might represent a decompensated response to METH-induced oxidative stress, neuroinflammation, and neuronal apoptosis.

Luteolin is a natural flavonoid compound widely present in medicinal plants [47,48]. Mounting evidence has proven that luteolin has antioxidative and anti-inflammatory properties, alleviating various kinds of diseases, such as inflammatory bowel disease [49], cancers [50], and brain injuries [36]. In the context of METH misuse-associated disorders, previous studies have revealed that luteolin prevents METH-induced neurotoxicity [51] and hepatotoxicity [52] by inhibiting apoptosis, autophagy, and inflammation. We found that the luteolin pretreatment could partially alleviate METH withdrawal-induced negative emotions and cognitive deficits (episodic memory in the NORT, but not spatial memory in the Y-maze). Moreover, luteolin prevented the prolonged METH withdrawal-induced abnormal activation of the PFC and CPu, as well as the downregulation of AKT/HO-1 signaling. The activation of AKT/HO-1 signaling has been shown to exert anti-apoptotic and antioxidant effects [53,54]. Thus, we suggest that luteolin may relieve affective and cognitive dysfunctions during METH withdrawal by suppressing METH-induced oxidative injuries and neuronal apoptosis in the PFC and CPu through the activation of AKT/HO-1 signaling.

FOXO1 is a pro-apoptotic and pro-inflammation transcription factor. The overexpression of FOXO-1 in macrophages has been shown to increase apoptosis and pro-inflammatory cytokine expression, such as that of IL-1beta [55]. The nuclear translocation of FOXO1 accelerates pro-apoptotic gene expression and causes neuronal death [56]. The inhibition of FOXO1 could attenuate copper-induced apoptosis in neural stem cells [57] and chidamide-triggered pyroptosis in T lymphoblasts [58]. Moreover, the FOXO1 inhibitor suppresses the mevastatin-induced increase in HO-1 expression in cardiac fibroblasts and attenuates the anti-inflammatory and antioxidative effects of mevastatin [59]. Unfortunately, few studies to date have revealed the role of FOXO1 in addictive substance use disorders. Our findings demonstrate that METH withdrawal increases, but luteolin decreases, the nuclear translocation of FOXO1. This may be one of the potential mechanisms underlying the protective effects of luteolin on the alleviation of METH withdrawal-induced neuronal apoptosis and oxidative injuries, as well as negative emotions and cognitive deficits.

Our study has several limitations. First, we only investigated the neuronal activity and AKT/FOXO1/HO-1 expression in two representative brain regions (the PFC and CPu) in METH withdrawal-induced negative emotions and cognitive deficits. The sub-regional functions of these anatomically and functionally heterogeneous brain regions and their projection relationships, and interactions with different kinds of neurons—as well as other molecular cascades in other brain regions in the context of METH withdrawal related cognitive and affective disorders still need to be uncovered. Second, we could not verify the specificity of the neuronal activity and AKT/FOXO1/HO-1 signaling changes to the negative emotions and cognitive deficits. Third, although the potential molecular mechanisms underlying the protective effects of luteolin on METH withdrawal-induced negative emotions and cognitive deficits have been preliminarily revealed in our study, the detailed and precise molecular cascades and the cellular and synaptic plasticity mechanisms were not fully elucidated. Additionally, we pretreated mice with luteolin before exposing them to METH in the present study. It would be better to administer luteolin after METH exposure from the view of translational relevance. Therefore, caution should be taken when interpreting the effects of luteolin on alleviating METH withdrawal-induced negative emotions and cognitive deficits. These aspects also provide directions for future research to further explore the in-depth mechanisms and potential invention strategies for METH-associated health issues.

In summary, our results demonstrate that prolonged METH withdrawal increases neuronal activity and suppresses AKT/HO-1 signaling in the PFC and CPu, potentially contributing to negative emotions and cognitive deficits. Luteolin pretreatment may mitigate these effects by normalizing neuronal activity and restoring AKT/HO-1 expression. Furthermore, luteolin reduces the nuclear translocation of FOXO1. These findings suggest that the potentially protective effects of luteolin on METH withdrawal-induced affective and cognitive dysfunctions may stem from attenuating METH-induced oxidative stress and neuronal apoptosis through the AKT/FOXO1/HO-1 pathway. Our results highlight the therapeutic potential of luteolin and its derivatives for treating METH-associated neurological disorders.

## 4. Materials and Methods

### 4.1. Animals

A total of 60 male C57BL/6 J mice (8 weeks old) were purchased from Vital River Laboratory Animal Technology (Beijing, China). The mice were housed in cages in groups of four with water and standard chow available ad libitum in a controlled environment (temperature of 20–24 °C, humidity of 40–60%, and a 12/12 h light/dark cycle). All mice were acclimatized to the environment for one week prior to the experiments to avoid the effects of stress. Our study protocols adhered to the Guidelines for the Care and Use of Laboratory Animals issued by the National Institutes of Health (NIH, Bethesda, MD, USA). Ethical approval for our study was obtained from the Institutional Animal Care and Use Committee of Xi’an Jiaotong University.

### 4.2. Reagents and Drug Administration Procedure

Methamphetamine hydrochloride powder (China Pharmaceutical and Biological Products, Beijing, China, 99.9% purity) was dissolved in 0.9% Sal. Luteolin (Med Chem Express, Monmouth Junction, NJ, USA) was dissolved in 0.9% Sal with 5% dimethyl sulfoxide (DMSO, Solarbio, Beijing, China), 20% polyethylene glycol 300 (PEG300, Solarbio, Beijing, China), and 2.5% polysorbate 80 (TWEEN 80, Solarbio, Beijing, China) to promote dissolution. The dose of METH was 3 mg/kg and that of luteolin was 20 mg/kg, based on previous studies [60,61]. All drugs were freshly prepared before the experiments and administered via intraperitoneal (i.p.) injection. To investigate the effects of luteolin on METH withdrawal-induced affective and cognitive dysfunctions, mice were randomly divided into four groups (*n* = 8 per group) and received one daily injection (i.p.) of Veh + Sal, luteolin + Sal, Veh + METH, or luteolin + METH. Luteolin was administrated 30 min before METH injections.

### 4.3. Behavioral Tests

#### 4.3.1. Anxiety-like Behaviors

(1) Open field test (OFT)

Mice were placed into the OFT chambers (43 cm length × 43 cm width × 43 cm height) to explore freely for 30 min, and their locomotion trajectories were recorded. The total distance traveled, central area distance traveled, and time spent in the central area for each mouse were recorded using Smart 3.0 software (Panlab Technology for Bioresearch, Barcelona, Spain).

(2) Elevated plus maze (EPM) test

The EPM apparatus consisted of a central zone (6 cm length× 6 cm width × 6 cm height), two open arms (33 cm length × 6 cm width), and two closed arms (33 cm length × 6 cm width × 6 cm height). Mice were placed into the central zone. Then, the time spent in each arm and the arm entry times were recorded for 5 min using Smart 3.0 software. The percentage of time spent in the open arm was calculated as the open arm time/(open arm time + closed arm time).

#### 4.3.2. Cognitive Function Detection

(1) Novel object recognition task (NORT)

The NORT consisted of 3 phases, including the adaptation phase (day 1–2), learning phase (day 3), and test phase (day 3–4). The adaptation phase lasted for 2 days, during which mice were placed in the NORT chambers (30 cm length × 20 cm width × 30 cm height) for 10 min to adapt to the apparatus. In the learning phase, two identical objects were put into the chambers 10 cm apart from each other and the side walls. Mice were placed into the chambers to explore for 5 min each time, for 3 times in total with a 15 min interval between each exploration. Three and twenty-four hours, respectively, after the end of the learning phase, one of the above same objects was replaced with a novel one. Then, mice were placed into the chambers to explore for 5 min and the time spent accessing each object was recorded with Smart3.0 software. The recognition index was calculated as the novel object exploring time/(novel object exploring time + familiar object exploring time).

(2) Y-maze test

The Y-maze apparatus consisted of 3 equal arms (30 cm length × 6 cm width × 15 cm height) with an angle of 120°. Mice were placed in the center area and allowed to explore for 5 min. Entering the 3 arms consecutively was defined as a successful alternation. The percentage of alternation was calculated as the number of successful alternations/(total arm entries − 2).

#### 4.3.3. Depressive-like Behaviors

(1) Sucrose preference test (SPT)

The SPT consisted of 2 phases, including the training phase (days 1–3) and test phase (day 5). During the training phase on day 1, mice were put into cages separately for 24 h, with 2 bottles of 1% sucrose water and free food. On days 2–3, one of the 1% sucrose water bottles was replaced with a bottle of normal drinking water, and the positions of sucrose water and drinking water were exchanged at the 24th hour point to avoid position preference. After the training phase, the mice were deprived of water and food for 24 h. On day 5, the mice were given one bottle of 1% sucrose water and one bottle of drinking water for 24 h, and the positions of the two bottles were exchanged in the middle of the test. The weight loss of the two bottles was recorded. The sucrose preference ratio was calculated as the sucrose consumption/(sucrose consumption + normal water consumption).

(2) Forced swim test (FST)

The FST apparatus consisted of several cylindrical transparent tanks (40 cm height × 25 cm diameter). During the FST period, each tank was filled to 30 cm high with water, with a water temperature of 25–27 °C. The total immobile time during the 6 min test was recorded using Smart3.0 software.

### 4.4. Sample Preparation and Western Blotting

After the behavioral tests, mice were anesthetized with isoflurane and immediately decapitated, and the PFC and CPu regions of the brain were separated on ice. The total protein samples were added with RIPA lysis buffer (Beyotime, Shanghai, China) with 1 mM phenylmethylsulfonyl fluoride (PMSF, Beyotime, Shanghai, China) and 1 mM phosphatase inhibitor, then ultrasonicated to extract the proteins. After centrifuging at 12,000× *g* for 15 min at 4 °C, the supernatants were collected and mixed with 5 × loading buffer and then boiled for 5 min. The nuclear and cytoplasmic samples were processed following the instructions provided in the kit (Beyotime, Shanghai, China). The protein concentration was measured using a bicinchoninic acid (BCA) assay (Solarbio, Beijing, China). Equal amounts of protein (20 μg) were loaded onto 10% sodium dodecyl sulfate–polyacrylamide gel electrophoresis (SDS-PAGE) and then transferred onto 0.22 μm polyvinylidene fluoride (PVDF) membranes (Millipore, Darmstadt, Germany). The membranes were blocked with Protein Free Rapid Blocking Buffer (Epizyme, Shanghai, China) at room temperature for 15 min. Next, the PVDF membranes were incubated with a primary antibody at 4 °C overnight and washed with phosphate-buffered Sal with 0.1% Tween-20 (1 × PBST) three times (10 min per wash) after incubation. After washing, the membranes were incubated with a secondary antibody at room temperature for 2 h. Finally, an enhanced chemiluminescence (ECL) kit (Mishushengwu, Xi’an, China) was used to detect the protein. The expression intensities of the protein bands were analyzed using ImageJ software (Version 1.53c, NIH, Bethesda, MD, USA). The primary antibodies used in this study were: p-AKT (Thr 308, 1:1000, Cell Signaling Technology, Boston, MA, USA), AKT (1:2000, Cell Signaling Technology, Boston, MA, USA), HO-1 (1:2000, Proteintech, Wuhan, China), FOXO1 (1:2000, Proteintech, Wuhan, China), PCNA (1:2000, Proteintech, Wuhan, China), and β-actin (1:5000, Proteintech, Wuhan, China).

### 4.5. Sample Preparation and Immunofluorescence

Mice were anesthetized with 5% sodium pentobarbital, and then transcardially perfused with 0.9% Sal, followed by 4% paraformaldehyde. Their brains were collected and post-fixed in 4% paraformaldehyde for 24 h. Then, the brains were removed into a 30% sucrose solution dissolved in 0.2 M phosphate buffer (PB, pH 7.4) for 48 h of dehydration. The brains were embedded in an optimal-cutting-temperature compound (OCT, Sakura, Torrance, CA, USA), and cut into 20 μm thick coronal sections on microtome (Leica, Wetzlar, Germany). The sections were washed 3 times (5 min per wash) with 1 × PBS, and then blocked in 5% goat serum solution (Boster, Wuhan, China) with 0.3% Triton X-100 (Servicebio, Wuhan, China) for 2 h at room temperature. Following this, the sections were incubated with c-Fos (1:400, Cell Signaling Technology, Boston, MA, USA) and FOXO1 (1:200, Proteintech, Wuhan, China) overnight at 4 °C. After washing, the sections were incubated with a secondary antibody (1:400, Proteintech, Wuhan, China) for 2 h at room temperature, and then washed and mounted for observation. AntiFade mounting medium (with DAPI) (Servicebio, Beijing, China) was used to observe the nucleus of the cells. All sections were observed using a fluorescence microscope (Zeiss, Oberkochen, Germany). The c-Fos positive cells were quantified using ImageJ software, with consistent parameter settings applied across all groups. The colocalization analysis and visualization were performed using the colocalization and scatter J tools in ImageJ software.

### 4.6. Statistical Analysis

GraphPad Prism (version 9.0) was used for all statistical analyses. All data are presented as means ± SEMs. The Shapiro–Wilk and Bartlett’s tests were used for the normal distribution and homogeneity of variance tests. All the data were normally distributed. Then, data were analyzed using Student’s *t*-test (two group comparisons) or two-way ANOVA (four group comparisons), accordingly. All post hoc pairwise comparisons were performed using the Bonferroni test. *p* values < 0.05 were considered statistically significant.

## Data Availability

All data in the present study are available from the corresponding authors upon reasonable request.

## Abbreviations

The following abbreviations are used in this manuscript:

BBB	blood–brain barrier
PFC	prefrontal cortex
CPu	caudate putamen
AKT	protein kinase B
FOXO1	forkhead box protein 1
HO-1	heme-oxygenase-1
PI3K	phosphoinositide 3-kinase
EPM	elevated plus maze
OFT	open field test
NORT	novel object recognition task
SPT	sucrose preference test
FST	forced swim test
